# Fragment-based drug discovery of small molecule ligands for the human chemokine CCL28

**DOI:** 10.1016/j.slasd.2023.02.004

**Published:** 2023-02-23

**Authors:** Angela L. Zhou, Davin R. Jensen, Francis C. Peterson, Monica A. Thomas, Roman R. Schlimgen, Michael B. Dwinell, Brian C. Smith, Brian F. Volkman

**Affiliations:** a Department of Biochemistry, Medical College of Wisconsin 8701 Watertown Plank Road, Milwaukee, WI 53226, USA; b Program in Chemical Biology, Medical College of Wisconsin 8701 Watertown Plank Road, Milwaukee, WI 53226, USA; c Department of Microbiology and Immunology, Medical College of Wisconsin 8701 Watertown Plank Road, Milwaukee, WI 53226, USA; d Center for Immunology, Medical College of Wisconsin 8701 Watertown Plank Road, Milwaukee, WI 53226, USA

**Keywords:** CCL28, NMR, Chemokine, Fragment, Drug discovery, Sulfotyrosine

## Abstract

The mucosal chemokine CCL28 is a promising target for immunotherapy drug development due to its elevated expression level in epithelial cells and critical role in creating and maintaining an immunosuppressive tumor microenvironment. Using sulfotyrosine as a probe, NMR chemical shift mapping identified a potential receptor-binding hotspot on the human CCL28 surface. CCL28 was screened against 2,678 commercially available chemical fragments by 2D NMR, yielding thirteen verified hits. Computational docking predicted that two fragments could occupy adjoining subsites within the sulfotyrosine recognition cleft. Dual NMR titrations confirmed their ability to bind CCL28 simultaneously, thereby validating an initial fragment pair for linking and merging strategies to design high-potency CCL28 inhibitors.

## 1. Introduction

CCL28, also called mucosae-associated epithelial chemokine (MEC), is a chemokine expressed in most mucosal epithelial tissues and is essential in multiple immune response pathways, especially in the regulation of cytokine-induced inflammation [1,2]. We previously solved the NMR structure of CCL28 [3], which adopts the chemokine fold, comprised of a conserved ‘N-loop’ that makes important receptor contacts, a three-stranded antiparallel *β*-sheet stabilized by two conserved disulfide bonds and an *α*-helix. In the CCL28 structure, the N-loop consists of residues 13–19 that connect the conserved ‘CC’ motif (C11 and C12) to a short helix (residues 21–26) that precedes the *β*1 strand. CCL28 also includes an unusual long C-terminal extension and third disulfide bond linking cysteines at positions 30 and 80.

**Figure 1.**
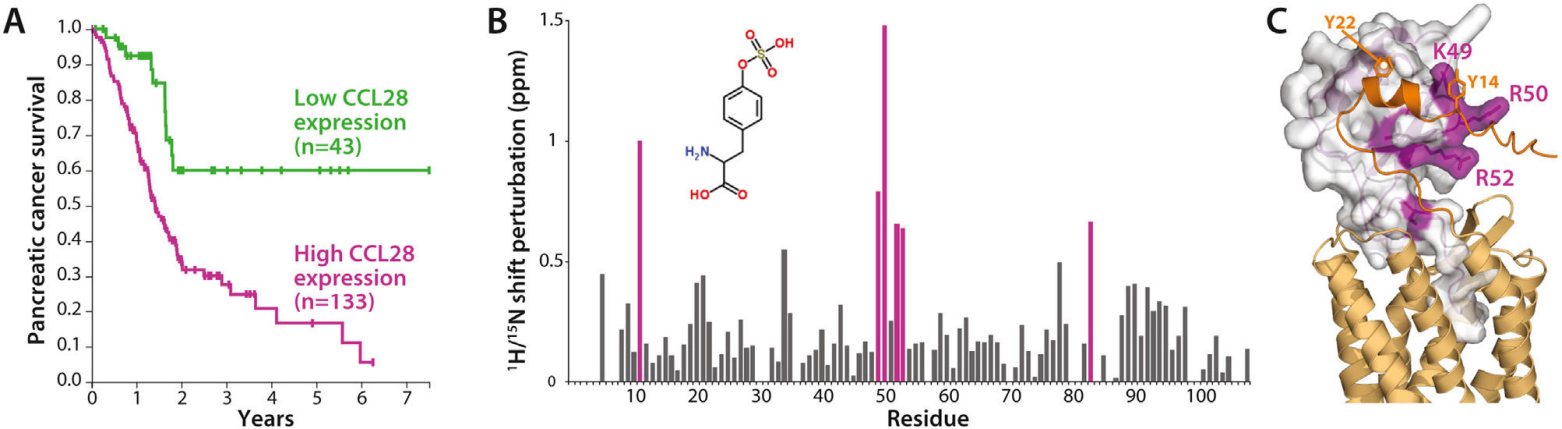
Validation of CCL28 as a cancer drug target. (**A**) CCL28 expression level (expression cutoff 2.7 FPKM; Human Protein Atlas, proteinatlas.org) is a prognostic indicator (P = 0.00014) for survival in pancreatic cancer. (**B**) Amide NH chemical shift perturbations measured by comparing ^1^H-^15^N 2D HSQC spectra of human CCL28 in the presence and absence of 100 mM sulfotyrosine. (**C**) Large (> 0.6 ppm) sulfotyrosine-induced shift perturbations highlighted (purple) on a model of the CCL28-CCR10 complex generated using Alpha Fold 2.

Despite serving as an essential component of the immune system, CCL28 plays a pro-tumoral role in several cancers [4]. While CCL28 has both anti- and pro-tumor properties, the latter dominate in the hypoxic cancerous Tumor Micro-Environment (TME) and involves stimulating the chemotaxis of myofibroblasts and various lymphocyte types, including Tregs, to the tumor site via CCR10 activation [4-7]. Tregs limit anti-tumor immune responses by suppressing the activation and proliferation of CD4+ helper T cells and CD8+ cytotoxic T cells while simultaneously promoting angiogenesis. Stromal myofibroblasts strengthen the extracellular matrix that blocks out antitumor drugs and immune cells [8]. Consistent with its tumorigenic functions, CCL28 expression levels from the Human Protein Atlas [9] are negatively correlated with survival rate in two of the three leading causes of cancer death in the United States [10], including pancreatic ductal adenocarcinoma (PDAC) [11,12] (Fig. 1A) and lung cancer [13]. In pancreatic cancer, *in vitro* CCL28 knockdown suppressed intrinsic PDAC cell proliferation, while CCL28 downregulation *in vivo* promoted the extrinsic upregulation of cytotoxic proteins, including perforin and the decreased infiltration of pancreatic stellate cells (PSCs) and blood vessel formation [11]. Recent findings have also positively correlated overexpression of CCL28 expression with enhanced tumor growth and migration in breast cancer [14].

Development of a small molecule inhibitor for CCL28 faces two main hurdles: first, small proteins like chemokines (~10 kDa) are traditionally considered poor targets for drug development, since they typically lack a deep hydrophobic active site or binding pocket. However, the accidental discovery of a high-affinity inhibitor that binds a relatively shallow site on the interleukin-2 surface expanded the range of potentially ‘druggable’ proteins to include cytokines, chemokines, and other small proteins and domains [15,16]. Chemokines have since been found to harbor specific sites for ligand binding, which can be identified using sulfotyrosine as a probe in NMR titrations (Fig. 1B) [17-21]. Second, chemokines engage their receptors by forming an extensive protein-protein interface (PPI). Most PPIs are difficult to disrupt with small molecules because the binding energy is distributed across a broad surface area. However, fragment-based drug discovery (FBDD), pioneered by Stephen Fesik at AbbVie in the 1990s [22], has yielded drugs in clinical trials [23]. Venetoclax, which received FDA approval in 2016 for chronic lymphocytic leukemia, emerged from an AbbVie NMR fragment screening program to treat cancer by disrupting anti-apoptotic Bcl-2 complexes [24].

Drawing upon these discoveries, we previously formulated a chemokine drug development strategy that targets a conserved sulfotyrosine recognition site representing an energetic hotspot within the chemokine-receptor interface [17,18]. The objective of this study, therefore, is to use our NMR screening pipeline to identify chemical fragments that may be used in the synthesis of a small-molecule inhibitor that could prevent CCL28 from binding to CCR10, and subsequently deter the development and proliferation of the tumorigenic microenvironment.

## 2. Materials and methods

### 2.1. AlphaFold modeling

Molecular modeling of the CCR10-CCL28 complex was generated with AlphaFold 2 [25] using the ColabFold platform on Google Colaboratory [26]. Human CCR10 and CCL28 sequences were obtained from UniProt (P46092 and Q9NRJ3). The human CCL28 gene encodes a 127 amino acid preprotein, and the UniProt entry is annotated with an *N*-terminal signal peptide corresponding to residues 1-19. Signal peptide cleavage would yield a 108-residue secreted protein in which the *N*-terminal sequence SEAILPIASS precedes the conserved CC motif, which we designate CCL28(1-108). Commercial sources of recombinant CCL28 supply either CCL28(1-108) or a truncated CCL28(4-108) version described as the native secreted form of human CCL28 by Pan *et al*. [27]. Our functional comparisons indicate that CCL28(4-108) is ~10-fold more potent as a CCR10 agonist than the longer version (MA Thomas and BF Volkman, unpublished results). Consequently, the sequences of human CCR10 and human CCL28(4-108) were submitted to ColabFold in hetero-oligomer format, and the program was run without structural templates to enhance output heterogeneity. The sequence alignment used MMseqs2 and UniRef90, and the value of pLDDT was used to rank the five resulting models against one another. The top-ranked model was manually inspected before placement into a synthetic biological membrane containing cholesterol, POPA, DDPC, DOPC, POPE, and POPS in a 6:2:2:8:6:1 ratio using Charmm-GUI [28-33]. Water, along with sodium and chloride ions at final concentrations of 150 mM, was then added to the system. After assembly, the system was downloaded in GROMACS format. The resulting model underwent 6 rounds of equilibration at 310 K before a 1 μs all-atom molecular dynamics (MD) simulation using GROMACS 2021.2 performed on the MCW Research Computing Center GPU server [34,35]. A frame at the simulation midpoint (500 ns) was employed to visualize the CCL28-CCR10 interface.

### 2.2. Protein purification

Uniformly ^15^N-labeled CCL28(1-108) and CCL28(4-108) proteins were expressed using a previously described SUMO fusion construct [36]. The pET28-Smt-CCL28 expression vector was transformed into *E. coli* strain BL21(DE3) and cells were grown at 37°C in M9 medium with ^15^N ammonium chloride. At a culture density of 0.7 absorbance units at 600 nm, protein expression was induced by adding 1 mM isopropyl-*β*-D-thiogalactopyranoside. After incubation at 20°C overnight, cells were pelleted at 5000 × g and stored at −80°C until further processing. Cell pellets were resuspended in 10 mL of a buffer containing 50 mM Na_2_PO_4_ (pH 8.0), 300 mM NaCl, 10 mM imidazole, 10 μL of Bacterial ProteaseArrest (G Biosciences), 0.1% v/v 2-mercaptoethanol (*β*ME), 100 μg lysozyme, and 5 units of DNase I (Thermo Scientific). Resuspended cells were lysed in two rounds of sonication on 50% duty cycle and 70% power. Inclusion bodies were collected by centrifugation at 15,000 × g. The insoluble inclusion body pellet was dissolved in buffer AD (6 M guanidinium chloride, 50 mM Na_2_PO_4_ (pH 8.0), 300 mM NaCl, 10 mM imidazole, 0.02% w/v sodium azide) and 0.1% v/v *β*ME. Cell membrane and debris were precipitated out by centrifugation at 15,000 × g.

The supernatant containing Smt-CCL28 was batch loaded onto 4 mL of His60 Ni Superflow Resin (TaKaRa). After 30 min incubation at room temperature, the column was washed with 4 × 10 mL of buffer AD with 0.1% v/v *β*ME, followed by elution with a buffer containing 6 M guanidinium chloride, 50 mM sodium acetate (pH 4.5), 300 mM NaCl, 10 mM imidazole, and 0.02% w/v sodium azide. All eluate samples were combined and refolded overnight via dropwise dilution into 100 mM Tris (pH 8.0), 5 mM cysteine, and 1 mM cystine buffer. Refolded Smt-CCL28 was concentrated via ultrafiltration (MWCO 10 kDa), and the tag was cleaved by incubation with Ulp1 protease at 4°C overnight. Cation-exchange chromatography was used for tag separation from the mixture. Samples were loaded onto SP Sepharose Fast Flow resin (GE Healthcare UK Ltd, Buckinghamshire, England) and washed using Tris (pH 8.0), 50 mM NaCl to remove the Smt-tag. A gradient of 50 mM to 1 M NaCl was used to elute the CCL28. Finally, samples were purified to >98% homogeneity using reverse-phase HPLC with a 30 min gradient from 20% to 40% acetonitrile in aqueous 0.1% TFA. CCL28 was frozen, lyophilized, and stored at −20°C. Mass spectrometry and nuclear magnetic resonance (NMR) spectroscopy verified purity, identity, and molecular weight.

### 2.3. NMR spectroscopy

NMR samples for fragment screening and titration experiments were prepared using a liquid handling robot designed for and dedicated to sample mixing and loading of NMR sample tubes as previously described [37,38], except that 1.7 mm sample tubes were used with sample volumes of 60 μL. All ^1^H,^15^N HSQC spectra were acquired at 25 °C and 600 MHz on a Bruker Avance III HD NMR spectrometer equipped with a 1.7 mm TCI CryoProbe and SampleJet sample handling robot. NMR data processing was performed in an automated manner, as previously described [37,38]. Custom Python scripts were used to automate the movement of NMRPipe processing scripts to each experiment directory, run the NMRPipe processing scripts, transfer transformed spectra to a processed data directory, compute HSQC difference spectra, generate HSQC overlay and HSQC difference contour plot files for each sample, perform principal component analysis calculations and K-means cluster analysis on all spectra acquired in each round of fragment screening.

### 2.4. Sulfotyrosine titration

A series of ^1^H,^15^N HSQC spectra was acquired on samples containing 50 μM ^15^N-CCL28(4-108) and either 1, 5, 10, 20, 50, 75, or 100 mM sulfotyrosine (sTyr). sTyr-induced peak movement was analyzed using NMRFAM-Sparky [39]. Combined ^1^H/^15^N chemical shift perturbations and *K*_d_ values were calculated as previously described [37].

### 2.5. Fragment identification by 2D NMR

Fragment screening was conducted against 50 μM ^15^N-CCL28(1-108) in an iterative multiplexed manner as previously described [37,38], except that each chemical fragment was present at a concentration of 500 μM instead of 1mM. A collection of 2,678 fragments comprised of the Core 1000 (Maybridge), Zenobia 1 (Zenobia), L5700 (Targetmol), and 3D Shape Diverse (Enamine) libraries were arranged into 12-plex groups for the first iteration. A consistent buffer was used across all NMR samples (50 mM dMES, 150 mM NaCl, 3% v/v DMSO, 0.02% w/v sodium azide, 5% v/v deuterium oxide) for 12-plex, 3-plex, and single compound screening rounds. Principal component analysis (PCA) and difference intensity analysis (DIA) comparisons of a reference (DMSO only) spectrum with each screening spectrum were used to identify potential hits, which were then verified by manual inspection of HSQC overlays and HSQC difference spectra, as previously described [37,38]. Candidate hits were repurchased and subjected to ^1^H-^15^N HSQC titration with 50 μM CCL28(1-108) at concentrations ranging from 0–6 mM. Repurchased compound identities were confirmed via 1D ^1^H NMR, and purity was determined to be at least 90% via natural abundance ^1^H-^13^C HSQC. For a subset of validated hits, CCL28 binding was measured in combination with a second fragment, each of which was titrated to a maximum concentration of 3 mM.

### 2.6. Computational fragment docking

Two validated hits (SPB07625 and CC10501) underwent computational docking onto the NMR structure of CCL28 (PDBID: 6CWS) using Schrodinger’s Maestro Glide [40]. To remove bias, a molecular grid was generated allowing ligands to dock within 35 Angstroms of the CCL28 centroid, encompassing the entire molecule. SPB07625 and CC10501 were independently prepared and docked onto CCL28 using a OPLS4 force field at pH 5 through pH 9. Glide ranked and visualized the top ten binding locations for each fragment on CCL28 along with their respective orientations. The top five hits for each molecule were inspected in PyMOL 2.4 (Schrödinger) to visualize proximity for potential linking.

## 3. Results

### 3.1. Identification of a potential CCL28-receptor binding hotspot

Sulfotyrosine modifications in the *N*-terminal extracellular domain of most chemokine receptors likely enhance binding to their cognate chemokine ligands [41]. As receptor-derived sulfopeptides and the free sulfotyrosine amino acid (sY) have been shown to bind several chemokines [17,42-48], we used sY as a probe to identify potential receptor binding hot spots on the CCL28 surface. A 2D NMR titration demonstrated that sY binding significantly perturbs CCL28 residues C11, K49, R50, R52, I53, and K83 (Fig. 1B). Homology modeling of the intact CCL28-CCR10 complex (Fig. 1C) places Y14 of the receptor *N*-terminus near K49, R50, R52, and I53, highlighting a potential sulfotyrosine recognition cleft between the N-loop and the *β*3 strand, analogous to those previously identified for CXCL12 [42,43] and CCL2 [49]. Together, the sulfotyrosine-induced chemical shift perturbations and homology modeling suggest that CCL28 harbors a ligand binding hotspot that could be targeted to develop small molecule inhibitors.

### 3.2. Chemical fragment screening of CCL28 by 2D NMR

**Figure 2.**
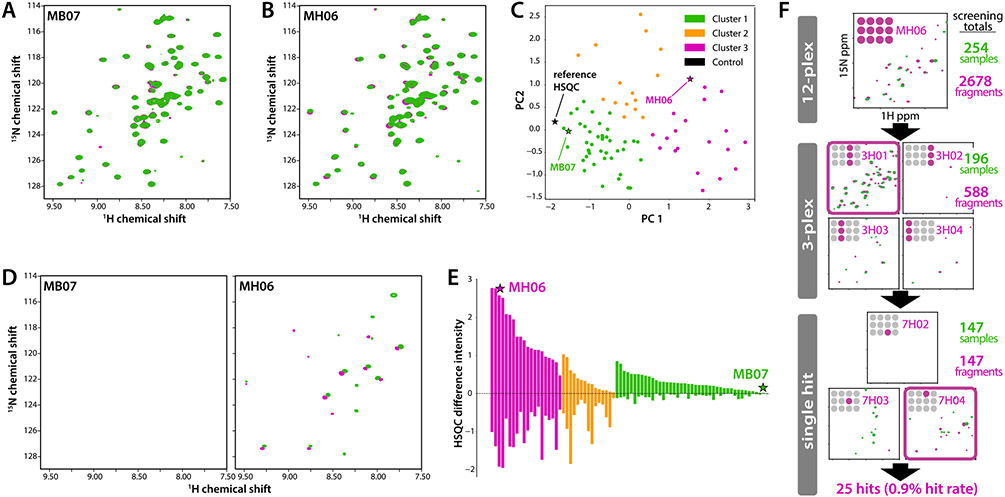
Fragment-based screening of CCL28 by 2D NMR. Overlays of CCL28(1-108) (100 μM) HSQC spectra in 3% v/v d_6_-DMSO (green contours) and a 12-plex mixture of compounds (0.5 mM each) from a Maybridge fragment library (magenta) for representative (**A)** non-hit (MB07) and (**B)** hit (MH06) samples. (**C)** PCA analysis of all 12-plex HSQC spectra, colored according to the result of k-means clustering. Hits are more likely found in cluster 2 or 3 than in cluster 1, which includes the reference spectrum (black star). (**D)** HSQC difference plots (reference spectrum is subtracted from the 12-plex spectrum) for non-hit and hit samples shown in panels (A) and (B). (**E)** DIA plot for all 12-plex samples in the Maybridge library screen, sorted by magnitude within k-means clusters from PCA analysis shown in panel C. (**F)** Summary of the primary screen of CCL28(1-108) against fragment libraries from Zenobia, Maybridge, Enamine and Targetmol, with a representative set of 12-plex, 3-plex and single compound HSQC difference plots shown for each sample leading to identification of an individual fragment hit (7H04).

CCL28(1-108) was screened against a collection of 2,678 chemical fragments assembled from multiple commercial sources. Applying a pipeline protocol we developed at the Medical College of Wisconsin [37,38], iterative multiplex (12-plex, 3-plex, single compound) screening by 2D NMR employed manual inspection of HSQC spectral overlays (Fig. 2A & B), PCA (Fig. 2C), HSQC difference spectra (Fig. 2D), and DIA with k-means clustering (Fig. 2E) to identify hits at each stage. HSQC analysis of single compounds yielded twenty-five candidate hits that induced significant CCL28 peak perturbations consistent with the patterns obtained for the corresponding 12-plex and 3-plex mixtures (Fig. 2F). Principal component analysis (PCA) of fragment-induced (1 mM) HSQC shift perturbations (data not shown) showed a cluster of 12 compounds that, in the HSQC titration, exhibit little or no evidence of specific, saturable binding to CCL28 (non-hits). Each of the 25 candidate hit compounds was repurchased from a different vendor for further validation.

### 3.3. Chemical fragment hit validation

**Figure 3.**
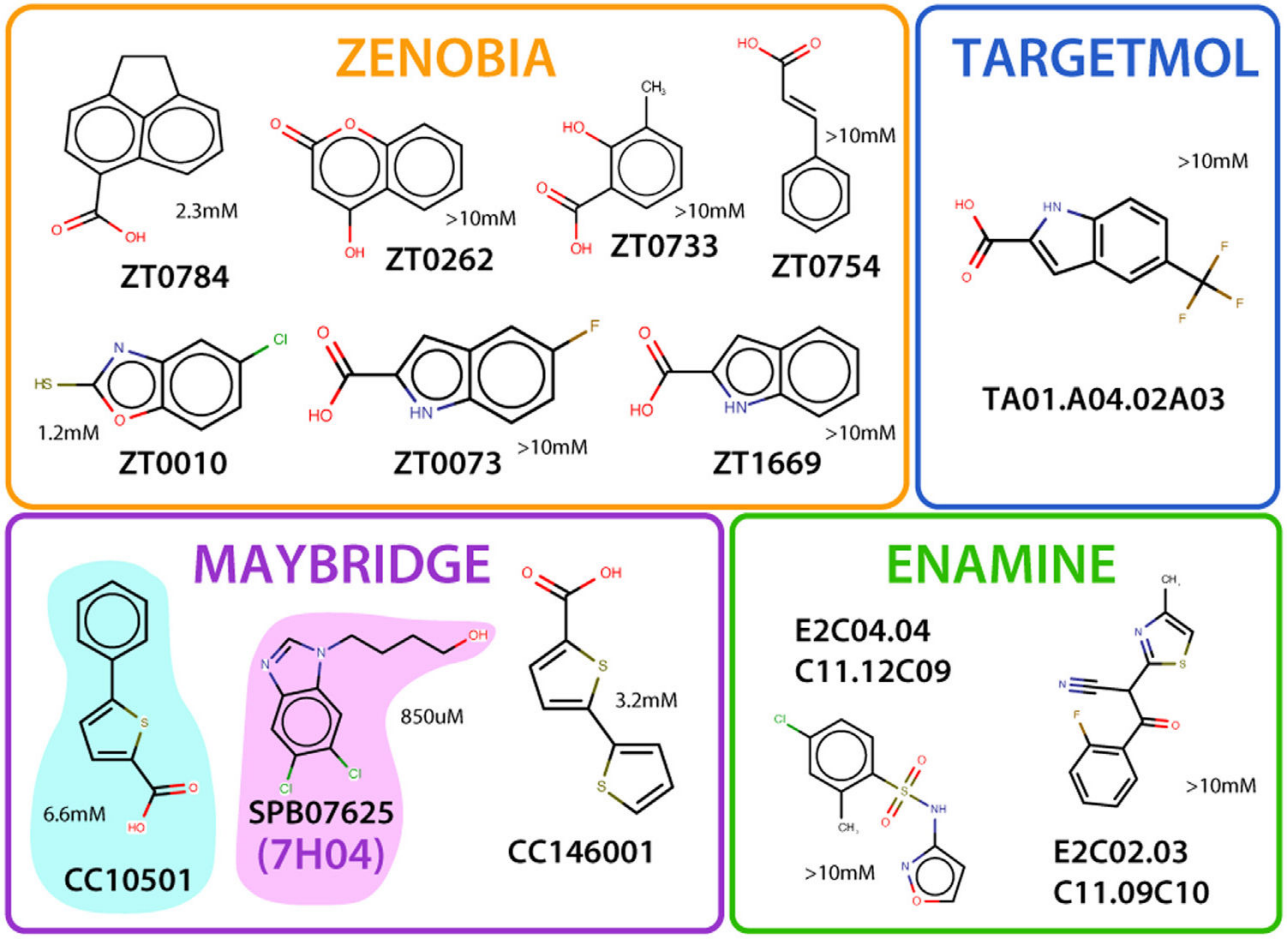
Fragment hits for CCL8. Final hits from Zenobia, Targetmol, Maybridge, and Enamine were determined through single NMR titration and PyMOL surface mapping onto CCL28, demonstrating specific binding with significant peak shifts not induced by a change in pH. *K*_d_ fitting of titration-derived chemical shift perturbation data for compounds SPB07625 (7H04 from Fig. 2) and CC10501 used in subsequent dual titration yielded values of 850 μM and 6.6 mM, respectively.

Because hits were identified in the primary screen from 2D NMR spectra acquired at a single fragment concentration, evaluating each candidate in terms of CCL28 binding affinity and specificity was essential. HSQC titrations up to a final concentration of 6 mM were performed using repurchased material for each candidate hit. Using the same chemical shift mapping approach employed for sTyr binding (Fig. 1B & C), titration-derived chemical shift perturbation data was used to ascertain and visualize the residues most affected by the addition of each compound. Fragment binding affinities were estimated by nonlinear fitting as previously described [50,51]. A total of 12 candidate fragments were judged non-hits because the pattern and concentration-dependence of shift perturbations were consistent with non-specific binding or were restricted to histidine residues suggesting a pH effect rather than compound binding. While *K*_d_ value estimates ranged from ~1 mM to > 10 mM (Fig. 3), the other 13 fragments reproduced HSQC perturbations observed in the primary NMR screen, and PCA clustered them in two groups distinct from the non-hits. Chemical shift mapping revealed that each cluster occupied one of two potential sites, implying the possibility of two distinct ligand-binding sites. Four fragments mainly affected residues S21, R21, E25, and the side chain of Q69 located in a cleft between the N-loop and *α*-helix. Another eight fragments largely perturbed residues in the sY binding cleft (Fig. 1C), including I53, which is analogous to V49 of CXCL12, a critical residue in facilitating binding interactions with sY21 of its receptor CXCR4 [18]. Compound ZT0784 perturbed residues located in both sites.

### 3.4. Dual sub-site binding of validated fragment hits

**Figure 4.**
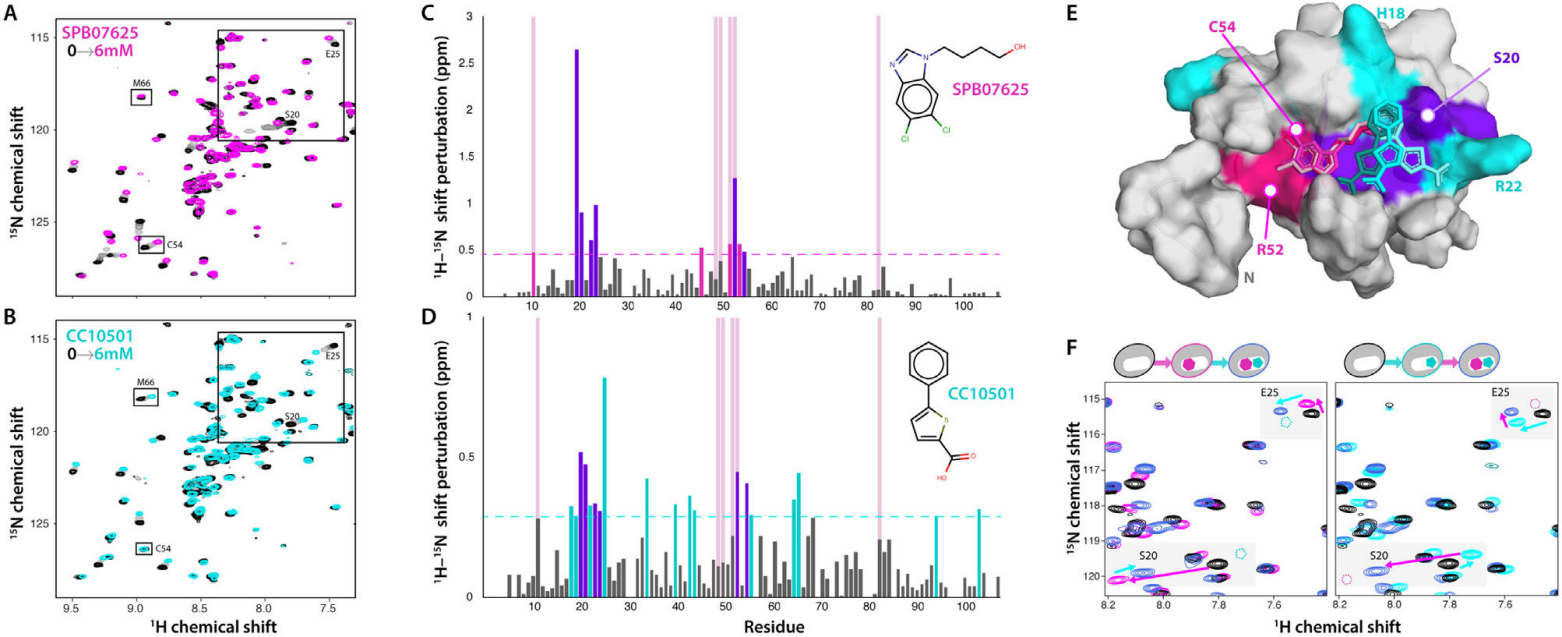
CCL28(4-108) upon titration with SPB07625 and CC10501. Overlays of CCL28(4-108) (50 μM) HSQC spectra in 3% v/v d_6_-DMSO (black contours) and single titrations up to 6 mM (lightening grayscale contours up to color) of Maybridge compounds (**A**) SPB07625 and (**B**) CC10501. Peaks that shift in response to only SPB07625 (C54) or CC10501 (M66) are boxed and labeled. (**C**) ^1^H/^15^N chemical shift perturbations induced by 6 mM SPB07625. Dashed line represents the threshold for residues represented in (E), chosen after computing the average and standard deviation of chemical shift perturbations for all residues. Light red full-length bars indicate the residues perturbed by the addition of sY. (**D**) ^1^H/^15^N chemical shift perturbations induced by 6 mM CC10501. (**E**) Residues with significant chemical shift perturbation from both compounds (violet) and those perturbed by only SPB07625 (magenta) and CC10501 (cyan) highlight a contiguous ligand binding site on the CCL28 NMR structure (unstructured *C*-terminal tail residues 81-108 not shown). Unbiased Glide docking of each fragment consistently positioned SPB07625 (magenta) and CC1050 (cyan) in adjoining subsites of the N-loop cleft closest to its compound-specific shift perturbations. Three of the top ten scoring positions of each compound are shown. (**F**) Dual-fragment NMR titrations (3 mM final concentrations) show that both compounds can bind CCL28 (50 μM) simultaneously, most clearly exemplified by residues S20 and E25. Final peak positions for S20 and E25 reflect the additive effects of perturbations from SPB07625 (magenta contours) binding followed by CC10501 (left panel) or vice versa (right panel), demonstrating identical net peak shifts (violet contours) regardless of the order of addition.

A key step in fragment-based drug discovery is the identification of multiple fragments that can bind adjoining subsites within a common cleft or pocket on the target protein. Patterns of HSQC peak shifts were compared to identify fragments that might be able to bind CCL28 simultaneously without displacing each other. In most cases, the patterns were too similar to determine if both compounds could bind simul-taneously. However, the perturbation profiles for one fragment pair, SPB07625 and CC10501, appeared to induce distinct sets of residues of the N-loop cleft and sY binding site that included a combination of fragment-specific and common peak shifts. Individual HSQC titrations of both fragments with CCL28(4-108) reproduced the patterns observed for CCL28(1-108) and the fragment-specific perturbation of C54 by SPB07625 (Fig. 4A) and M66 from CC10501 (Fig. 4B). Importantly, both induced a peak shift for S20 and E25 but in different directions, consistent with distinct binding modes for the two fragments. Shift perturbations induced by 6 mM of each fragment are plotted as a function of CCL28 residue in Figs. 4C & 4D. The largest SPB07625-induced shifts were observed for residues I53, S20, R21, and L24. In addition to I53, S20, and R21, CC10501 additionally yielded strong peak shifts for E25, Q69, and M66. The combined set of chemical shift perturbations highlights a contiguous CCL28 surface (Fig. 4E), with the middle of the N-loop cleft perturbed by both compounds (purple), while one end is affected only by SPB07625 (magenta) and CC10501-induced shifts are localized to the short helix at the other end (cyan). Unbiased computational docking of the individual fragments placed each within the cleft nearest to its corresponding fragment-specific shift perturbations (Fig. 4E).

Dual titrations were then performed in which one fragment (e.g. SPB07625) was titrated (0–3 mM) into a sample containing 3 mM of the other fragment (CC10501) and vice versa. HSQC overlays in Fig. 4F highlight the effect of serial titration of SPB07625 and CC10501 on the peaks for S20 and E25. In each case, the addition of both fragments induces CCL28 shift perturbations that correspond to the vector sum of the individual fragment-induced shifts, irrespective of the order of addition to the sample. This result suggests that SPB07625 and CC10501 can occupy adjoining subsites within the same cleft on the CCL28 surface.

## 4. Discussion

CCL28 fragment screening employed our semi-automated pipeline protocol initially validated on three different protein targets, including a chemokine [37]. The CCL28 hit rate of 0.9% from a collection of four commercial chemical fragment libraries (Fig. 2) was lower than the 2% hit rate obtained from CXCL12 screening against the Zenobia 1 library (352 compounds) [37]. Chemical redundancy, a less druggable target, or other unknown factors may contribute to the difference in overall hit rates. However, chemical shift mapping localized most of the 13 fragment hits (Fig. 3) to a conserved cleft bordered by the N-loop and *β*3-strand of CCL28 that contains the sulfotyrosine binding pocket identified by NMR (Fig. 1B).

Significant perturbations unique to sY include residues K49 and R50 and likely result from ionic interactions between the positively charged side chains and the negative charge of the sulfonated tyrosine. AlphaFold modeling positions Y14 of the CCR10 peptide in close vicinity of K49 and R50 (Fig. 1C), however, Liu *et al.* predicted that Y22 of the CCR10 *N*-terminus is sulfonated [52]. AlphaFold modeling places Y22 above the N-loop cleft where it could interact with many of the same residues perturbed by fragment binding. As both the model and predictions of sulfonation have not been confirmed with experimental data, it remains to be seen which of the tyrosine residues participates directly in CCL28-CCR10 recognition or interacts with the side chains of K49 and R50.

An NMR-based screen of 2,678 chemical fragments yielded 13 hits, two of which were selected for further detailed examination. As shown in Fig. 4E, computational docking consistently placed SPB07625 and CC10501 adjacent to each other within the N-loop cleft, in the vicinity of residues perturbed by sY binding, consistent with chemical shift mapping of each fragment binding site on the CCL28 surface. In comparison to CC10501 and SPB07625, titration of sY with CCL28 resulted in significant (chemical shift of at least two standard deviations above the mean) perturbation of residues C11 and R52 in common with SPB07625, and I53 in common with both compounds. R52 and I53 highlight a cleft analogous to the sY recognition site previously found on CXCL12, and computational docking also places SPB07625 at that site, indicating that SPB07625 and possibly other structurally similar fragments may compose the critical region of an eventual inhibitor molecule that will compete directly with CCR10 peptide for the sY recognition site on CCL28 (Fig. 4E). While CC10501 and SPB07625 alter the chemical environment of several of the same residues, there are no shared significant residue perturbations between CC10501 and sY. Consistently, highlighting of the residues most affected by CC10501 illuminates a region adjacent to the sY recognition site, and computation docking places CC10501 adjacent to SPB07625 without overlap. Together with conclusions from the dual titration data, we can speculate that CC10501 and structurally similar fragments may not occupy the sY recognition site but could block interactions with neighboring CCR10 residues and guide fragment growth, merging and linking strategies to increase the overall affinity of a novel small molecule ligand for CCL28.

Given the multiple mechanisms through which CCL28 can enhance tumor development by attracting plasma cells, Tregs, and myofibroblast-like stromal cells to the tumor site, and the numerous studies reporting the anti-tumor benefits of its downregulation, CCL28 is a promising therapeutic target whose inhibition could have significant implications on cancer patient survival [7]. Taken together, the overall agreement between our experimental data, docking calculations and homology modeling suggest that small molecules that bind a conserved cleft on the CCL28 surface could inhibit CCR10 binding, and that at least one pair of compounds identified in our NMR-based screen of 2,678 chemical fragments will be a suitable starting point for merging or linking strategies. Next steps include performing internal and external SAR analysis, design and synthesis of compounds joining SPB07625 and CC10501, and CCL28 binding studies of the resulting molecules. The ultimate goal is to develop a small molecule inhibitor that can block the tumor-directed trafficking of regulatory T cells, reduce the immunosuppressive nature of the tumor microenvironment in PDAC, and improve the efficacy of immunotherapies that are highly effective in other cancers.
